# Contactless Sleep Monitoring for the Detection of Exacerbations in People With Chronic Obstructive Pulmonary Disease: Protocol for a Longitudinal Observational Study

**DOI:** 10.2196/63230

**Published:** 2025-03-14

**Authors:** Julie Egmose, Thomas Kronborg, Ole Hejlesen, Stine Hangaard

**Affiliations:** 1 Department of Health Science and Technology Aalborg University Gistrup Denmark

**Keywords:** disease exacerbation, chronic obstructive pulmonary disease, contactless measurements, sleep monitoring systems, heart rate measurement, respiration rate measurement, radar technology, health literacy, patient remote monitoring

## Abstract

**Background:**

Exacerbations of chronic obstructive pulmonary disease (COPD) are one of the main causes of mortality, and early detection of exacerbations is thus essential. Telemedicine solutions have shown promising results for the detection of exacerbations in COPD and have increasingly been used. However, the effect of telemedicine is divergent. According to several studies, respiration rate (RR) increases before, during, and after an exacerbation and the change is measurable with several contactless devices. Despite this, RR is rarely measured, and telemedicine solutions only use wearable devices for measuring RR, even though wearable respiratory monitoring devices have been associated with certain drawbacks. Contactless devices are often used during sleep, as measurements conducted during sleep minimize the risk of disturbance from physical activities. However, the potential of measuring RR and heart rate (HR) during sleep for the detection of exacerbations in COPD remains unclear.

**Objective:**

The aim of this observational study is to investigate whether contactless measurement of RR, HR, and sleep stages can be used to detect exacerbations in people with COPD.

**Methods:**

An observational study including 50 participants with COPD will be conducted. The participants reside in Aalborg municipality, located in the North Denmark Region. Participants will use a contactless monitor (Sleepiz One+) near their bed during sleep for a period of 4 months. After data collection, descriptive statistics will be used to identify any extremes or variations in RR, HR, or sleep stages in the nights preceding an exacerbation. Correlation analysis will be performed to evaluate the relationship between the number of exacerbations and extremes or variations in RR, HR, or sleep stages. Finally, qualitative interviews will be conducted with 12 participants to explore their experiences of sleeping with the monitor nearby.

**Results:**

Recruitment started at the end of April 2024. A total of 12 participants have been recruited, and the remaining participants are expected to be recruited during March and April 2025. Six out of 12 participants have completed the data collection and qualitative interview stages. Overall data collection is expected to be completed by September 2025. The results are expected to provide insight into the potential for identifying extremes or variations in RR, HR, or sleep stages in the days preceding an exacerbation. Additionally, the results are expected to assess the correlation between the number of exacerbations and extremes or variations in RR, HR, and sleep stages.

**Conclusions:**

The findings from this study may clarify the possibility of using a contactless monitor to detect exacerbations in COPD. Furthermore, the results may have the potential to improve the ability to predict exacerbations in the future.

**International Registered Report Identifier (IRRID):**

DERR1-10.2196/63230

## Introduction

Chronic obstructive pulmonary disease (COPD) poses a global health challenge. In 2019, around 212 million people were diagnosed with COPD, and it was the third leading cause of death with approximately 3.3 million deaths [1,2]. COPD is associated with significant economic costs, which increase with the progression of the disease [1]. In the European Union, the direct expenses related to respiratory diseases are estimated to comprise approximately 6% of the total annual health care expenditures, with COPD accounting for 56% of respiratory disease expenditures [1].

Exacerbations in COPD are characterized by exacerbation of shortness of breath and/or coughing with increased sputum over a period of <14 days. These escalating symptoms may be accompanied by rapid breathing and/or rapid pulse [1,3]. Frequent exacerbations are associated with reduced quality of life (QoL), increased risk of rehospitalization, and higher mortality rates compared to fewer exacerbations [4]. Hence, it is essential to prevent exacerbations.

Timely treatment of exacerbation reduces the risk of hospitalization and increases QoL [5,6]. Identification of the first symptoms of exacerbations is therefore essential to initiate early treatment. For early detection of exacerbations, telemedicine solutions are increasingly used, which seem to have a positive effect on QoL and hospital admissions [7,8]. However, the effectiveness of telemedicine solutions in preventing exacerbations is not convincing, and the results are generally divergent, indicating the need for further research [7-11].

In telemedicine, monitoring of physiological parameters is widely used for early detection of exacerbations. Primarily, oxygen saturation and heart rate (HR) are measured and used for detection of exacerbations [11-14]. However, the telemedicine solutions are generally based on studies with a low methodological quality, highlighting the need for robust, well-designed clinical trials [14].

According to several studies, both respiration rate (RR) and HR increase before, during, and after an exacerbation [15-17]. Despite measurement of RR being more effective than HR for early detection of exacerbation, only a few telemedicine solutions include measurements of RR [11-14]. The lack of RR measurement may possibly be attributed to the general challenge of measuring RR over an extended time period at home [18]. Moreover, respiratory monitoring equipment has been associated with certain drawbacks such as discomfort and difficulty in use [19]. Overall, the usability of respiratory monitoring systems is sparsely investigated [18].

Various contactless devices are capable of measuring RR [20], including contactless radar-based monitors [21,22]. Existing telemedicine solutions only use wearable devices for RR measurements [19,23-25], even though it seems possible to measure a change in RR during exacerbation using contactless devices [26]. Contactless devices enable free movement and eliminate the discomfort associated with wearable devices. Discomfort can hinder measurement over an extended time period [22,27,28], and contactless devices are therefore increasingly used for measurements of physiological parameters [29]. However, contactless devices require the user to be near the device and are therefore often used for overnight measurements, where the user remains close to the device for an extended time period [20]. During sleep measurement, it is essential that the equipment is contactless to minimize the impact on sleep quality [30]. Sleep monitoring minimizes the risk of measurements being influenced by factors such as physical activity. Several contactless devices are capable of measuring both RR and HR [20-22]. To the best of our knowledge, no previous studies have conducted frequent contactless measurement of RR and HR during sleep for detecting exacerbations in people with COPD.

The overall objective of the study is to investigate whether physiological variables such as RR, HR, and sleep stages can be used to detect exacerbations in people with COPD. This study is based on the hypothesis that data from frequent measurement of RR and HR during sleep can be used to detect exacerbations in COPD. Ultimately, these data may improve the prediction of exacerbations in COPD.

## Methods

### Study Design

This study is a longitudinal observational study and is expected to run from April 2024 to September 2025 [31,32]. This study will recruit participants from the Danish telemedicine service, TeleCare Nord (TCN), which is part of routine care in the North Denmark Region [33]. The telemedicine service includes measurements of oxygen saturation, pulse, blood pressure, weight, and patient-reported outcomes once a week [34].

### Eligibility Criteria

#### Inclusion Criteria

People aged ≥18 years with a diagnosis of COPD will be included in the study, and both men and women are qualified. Moreover, people residing in the Aalborg Municipality will be deemed eligible to participate.

#### Exclusion Criteria

The exclusion criteria are the inability to complete questionnaires, pregnancy, nursing, electronic implants, and children or pets sleeping in the same bed as the participants, as it can disturb the measurements.

### Recruitment

**Figure 1:**
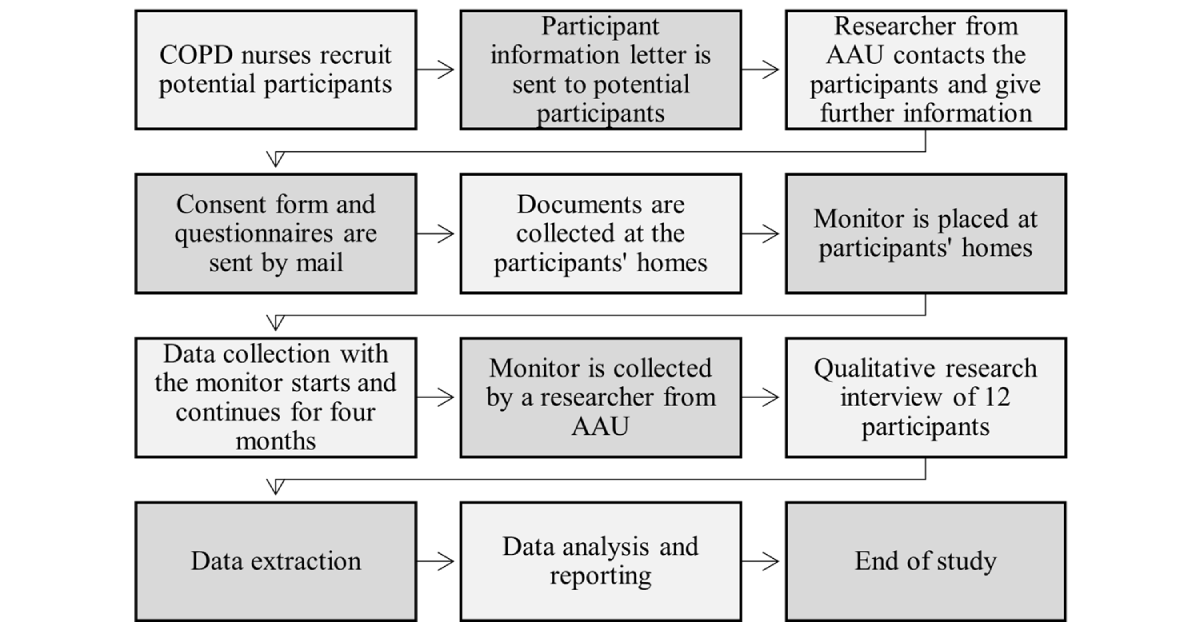
The study procedure from recruitment until the end of the study. AAU: Aalborg University; COPD: chronic obstructive pulmonary disease.

For the observational study, 50 participants will be recruited from Northern Jutland, Denmark. Initially, recruitment is conducted by specialized COPD community nurses (or a related clinician; Figure 1). The specialized COPD nurse gives a short introduction to the trial and in case of someone is interested in participating, a researcher from Aalborg University (AAU) will send a participant information letter by mail. Hereafter, the participants are contacted by a researcher from AAU who will provide further information about the study. If the individual is interested in participating, a consent form and questionnaires regarding baseline information will be sent by mail.

### Study Setting

After the participants have received and completed the questionnaires and consent forms, a researcher from AAU will collect the documents at the participants' homes. Subsequently, a contactless radar-based monitor (Sleepiz One+) will be placed near the participants’ bed. The monitor measures RR, HR, and sleep stages, including rapid-eye-movement sleep, light sleep, and deep sleep. The participants are asked to take measurements throughout the study period. The monitor measures changes in distance originating from breathing activity and heart contractions by using Doppler radar. These distance changes are analyzed using signal processing algorithms to extract RR and HR every fifth second.

The monitor is positioned on a stand 5-10 cm above mattress level, 40-50 cm from the participant pointing to the lower part of the chest. The monitor measures the participant during sleep and only measures the person who is positioned closest to the monitor. Therefore, the participants will be instructed to stay closest to the monitor during sleep, since it would disrupt the measurements if partners were sleeping closest to the monitor. Furthermore, participants will be instructed to turn on the monitor before going to sleep and turn it off upon waking throughout the entire study to ensure consistent data collection. This approach also prevents the monitor from recording data from partners who may go to bed earlier or stay in bed longer than the participants.

Neither the nurses nor the participants will have access to the data from the monitor during the study. Data from the monitor are solely used retrospectively to investigate whether it is possible to identify changes in RR, HR, or sleep stages preceding exacerbations. After the researcher from AAU had placed the monitor at the right position and ensured that the monitor works, data collection was carried out for 4 months.

The data will automatically be transmitted through Wi-Fi to a web application connected to the monitor, enabling data extraction. This web application allows users to view and analyze data collected by the contactless monitor. Participants will be registered as users in the web system by a researcher from AAU, who will also connect a monitor to each individual participant.

The questionnaires will be used to explore health status and health literacy. The health status involves baseline information (gender, age, civil status, educational level, deployment, years since COPD diagnosis, smoking status, and comorbidities), the COPD Assessment Test (CAT) [35], the Medical Research Council (MRC) dyspnea score [36], and The European Health Literacy Survey Questionnaire (HLS-EU-Q16) [37,38]. Additionally, the Danish Short Test of Functional Health Literacy in Adults (DS-TOFHLA) [39] questionnaire will be distributed to assess health literacy.

In cases of missing data for more than 2 consecutive nights, the participants will be contacted by a researcher from AAU by telephone to clarify the underlying reason and resolve that. Once data collection for 4 months (±10 nights) is completed, the monitor is collected by a researcher from AAU.

Data will be extracted from the web application connected to the monitor, TCN, and the National Patient Registry to determine the number and dates of exacerbations. Exacerbations are defined as hospitalizations caused by COPD or self-initiated treatment with antibiotics and/or steroids [40]. To obtain the exact dates of exacerbations, hospitalizations related to COPD will be extracted from the National Patient Registry. Similarly, to determine the dates of exacerbations related to self-initiated treatment, participants are already registering their use of antibiotics and steroids as part of TCN. If self-initiated treatment is registered, a researcher from AAU will contact the COPD nurse to obtain information about when the treatment began, thereby obtaining the exact date of the exacerbation.

### User Experience

A total of 12 participants will be asked to participate in a qualitative research interview, focusing on the experience and acceptance of sleeping with the monitor nearby over an extended period. The number of informants for the qualitative research interviews is expected to be sufficient, as data saturation is typically achieved with 12 participants [41]. However, the final number of informants may vary slightly, depending on when data saturation is achieved. To explore the participants’ perspectives on using the monitor, the qualitative research interview will be supplemented with the System Usability Scale (SUS) [42] to gather additional information regarding the usability of the system.

Interviews and surveys will be conducted after participants have used the monitor for at least 2 months, allowing them to gain a more realistic understanding of the long-term use.

Data collected during the interviews will be analyzed using the thematic analysis approach developed by Liamputtong [43] and Braun and Clarke [44]. The SUS score will be calculated in accordance with the guidelines presented by Lewis and Sauro [45]. The scores will be analyzed descriptively by using statistical tools in Excel (Microsoft 365 MSO; version 2406, build 16.0.17726.20222).

### Statistical Analysis

Overall, the data analysis will include descriptive statistics and logistic regression. Descriptive statistics will be used to calculate mean, SD, and variance for RR, HR, and the time spent in different sleep stages. Logistic regression is particularly relevant when working with binary outcomes to calculate the probability of an event occurring based on one or more independent variables. Since this study investigates whether changes in physiological parameters occur in the nights preceding an exacerbation, logistic regression is an appropriate method for analysis. Logistic regression will be used to calculate the probability of developing an exacerbation based on the descriptives derived from RR, HR, and sleep stages as independent variables. Odds ratios based on logistic regression and the *F* statistic (*P*<.05) will determine whether each descriptive is independently associated with developing an exacerbation. Furthermore, correlations between the descriptives will be examined.

### Sample Size

A sample size of 40 participants will provide 90% power to demonstrate a significant correlation coefficient of 0.5 between the number of exacerbations and extremes or variations in RR, HR, and sleep stages. Considering a presumed dropout rate of 25%, based on previous findings in a study among people affiliated with TCN (ClinicalTrials.gov NCT05218525), 50 participants need to be recruited in this study.

### Ethical Considerations

The study has been approved by the Regional Ethical Committee for Medical Research in the North Denmark Region (N-20230072). The study will be performed in accordance with the tenets of Helsinki Declaration [31] and the principles of Good Clinical Practice. Written consent will be obtained from each participant after detailed verbal and written information about the study has been given and will be obtained before inclusion in the study [31]. Data will be anonymized and stored securely in compliance with the Danish Data Protection Rules [32]. The protocol will not be modified unless a new approval from the Regional Ethical Committee is obtained. The participants will not be provided with any compensation for their participation. There are no expected risks associated with participation in the trial. There is no increased risk of exacerbations, as the participants are not exposed to any interventions that could contribute to the development of exacerbations in COPD.

## Results

The study received final approval from the Regional Ethical Committee for Medical Research in the North Denmark Region on April 10, 2024 (N-20230072). In April 2024, the first participant information letter was sent by mail to the initial potential participants. The first participant was recruited in May 2024. A total of 12 participants have been recruited so far. Six out of 12 participants have been completed. These participants have also participated in qualitative research interviews at the end. The delivery of the remaining monitors has been delayed and is expected to be underway in March and April 2025. The remaining participants will be recruited once the delayed monitors are received. Data collection will begin as participants are recruited. The first participant completed the data collection stage at the beginning of October 2024 and the last participant is expected to complete this stage by September 2025.

## Discussion

### Anticipated Findings

The main finding of this study is presumed to be a new way to detect COPD exacerbations using contactless sleep monitoring. The study aims to provide knowledge regarding the possibility of detecting COPD exacerbations through frequent measurements of RR, HR, and sleep stages collected by using a contactless monitor during sleep. If the study proves that exacerbations can be detected by contactless measurements, the results have the potential to improve the ability to predict exacerbations in the future. Predicting exacerbations enables the possibility of initiating early treatment, which is expected to reduce hospitalizations and increase the quality of life among people with COPD.

Additionally, the study is also expected to provide knowledge regarding the experience of using the monitor more permanently. This is considered valuable knowledge about the potential of the monitor, as users’ experience and acceptance are important to consider when developing and implementing new technologies [46,47].

An expected strength of this study is the high frequency of measurements conducted over an extended time period. To our knowledge, no previous studies have monitored RR by a contactless monitor with a high frequency of measurements over an extended time period in people with COPD. However, continuous data collection can be crucial in this study because missing data from the nights preceding an exacerbation can reduce the ability to detect exacerbations, as symptoms primarily change a few days prior to onset [48]. While the recommendation to turn the monitor on/off throughout the entire project period is expected to meet the participants’ needs regarding privacy in the bedroom, this recommendation can also increase the risk of missing data. Therefore, it is important to be aware of missing data throughout the study period.

Another expected strength is that the measurements are conducted during sleep without physically touching the participants. This is expected to eliminate the discomfort associated with wearable equipment. Moreover, conducting measurements during sleep reduces the risk of data being influenced by factors like physical activity, which can be challenging to control when people perform the measurements at home during the day on their own. Øverst på formularen

This study may be limited by the risk of observing too few exacerbations during the project period. To address this, most participants will collect data during the winter, when exacerbations are more frequent. However, participants will also collect data at different times of the year, and thus across different seasons, which will help increase the generalizability of the results. Additionally, to mitigate this potential limitation, exacerbations of varying severities will be included to increase the likelihood of capturing exacerbations. This approach was also chosen because it is of interest to examine whether it is possible to detect changes or patterns in the days preceding different severities of exacerbations. The study has the potential to improve the ability to detect COPD exacerbations and thereby initiate early treatment to reduce hospitalization.

### Dissemination Plans

The results are expected to be published in international scientific journals. All results will be reported anonymously. The results will be published irrespective of whether they are positive, negative, or inconclusive. The authorship will be admitted to persons who have participated to the design, interpretation, conduct, and reporting of the trial. The results will also be communicated to participants who have indicated that they wish to be informed.

## Abbreviations

AAU: Aalborg University
CAT: COPD Assessment Test
COPD: Chronic obstructive pulmonary disease
DS-TOFHLA: Danish Short Test of Functional Health Literacy in Adults
HLS-EU-Q16: The European Health Literacy Survey Questionnaire
HR: Heart rate
MRC: Medical Research Council dyspnea score
QoL: Quality of life
RR: Respiration rate
TCN: TeleCare Nord

## Data Availability

Data will not be made publicly available as the study participants have not consented and their data fall under General Data Protection Regulation regulations.

## References

[1] (2024). Global strategy for prevention, diagnosis and management of COPD: 2024 Report. Global Initiative for Chronic Obstructive Lung Disease.

[2] GBD 2019 Chronic Respiratory Diseases Collaborators (2023). Global burden of chronic respiratory diseases and risk factors, 1990-2019: an update from the Global Burden of Disease Study 2019. EClinicalMedicine.

[3] Celli BR, Fabbri LM, Aaron SD, Agusti A, Brook R, Criner GJ, Franssen FME, Humbert M, Hurst JR, O’Donnell D, Pantoni L, Papi A, Rodriguez-Roisin R, Sethi S, Torres A, Vogelmeier CF, Wedzicha JA (2021). An updated definition and severity classification of chronic obstructive pulmonary disease exacerbations: the Rome proposal. Am J Respir Crit Care Med.

[4] MacLeod M, Papi A, Contoli M, Beghé Bianca, Celli BR, Wedzicha JA, Fabbri LM (2021). Chronic obstructive pulmonary disease exacerbation fundamentals: diagnosis, treatment, prevention and disease impact. Respirology.

[5] Tomasic I, Tomasic N, Trobec R, Krpan M, Kelava T (2018). Continuous remote monitoring of COPD patients-justification and explanation of the requirements and a survey of the available technologies. Med Biol Eng Comput.

[6] Wilkinson TMA, Donaldson GC, Hurst JR, Seemungal TAR, Wedzicha JA (2004). Early therapy improves outcomes of exacerbations of chronic obstructive pulmonary disease. Am J Respir Crit Care Med.

[7] Hong Y, Lee SH (2019). Effectiveness of tele-monitoring by patient severity and intervention type in chronic obstructive pulmonary disease patients: A systematic review and meta-analysis. Int J Nurs Stud.

[8] Barbosa MT, Sousa CS, Morais-Almeida M, Simões Maria J, Mendes P (2020). Telemedicine in COPD: an overview by topics. COPD.

[9] Lippi L, Turco A, Folli A, D'Abrosca Francesco, Curci C, Mezian K, de Sire A, Invernizzi M (2023). Technological advances and digital solutions to improve quality of life in older adults with chronic obstructive pulmonary disease: a systematic review. Aging Clin Exp Res.

[10] Gregersen TL, Green A, Frausing E, Ringbæk Thomas, Brøndum Eva, Suppli Ulrik Charlotte (2016). Do telemedical interventions improve quality of life in patients with COPD? A systematic review. Int J Chron Obstruct Pulmon Dis.

[11] Jang S, Kim Y, Cho W (2021). A systematic review and meta-analysis of telemonitoring interventions on severe COPD exacerbations. Int J Environ Res Public Health.

[12] Sul A, Lyu D, Park D (2018). Effectiveness of telemonitoring versus usual care for chronic obstructive pulmonary disease: a systematic review and meta-analysis. J Telemed Telecare.

[13] Pedone C, Lelli D (2015). Systematic review of telemonitoring in COPD: an update. Pneumonol Alergol Pol.

[14] Al Rajeh A, Hurst J (2016). Monitoring of physiological parameters to predict exacerbations of chronic obstructive pulmonary disease (COPD): a systematic review. J Clin Med.

[15] Pinto-Plata VM, Livnat G, Girish M, Cabral H, Masdin P, Linacre P, Dew R, Kenney L, Celli BR (2007). Systemic cytokines, clinical and physiological changes in patients hospitalized for exacerbation of COPD. Chest.

[16] Parker CM, Voduc N, Aaron SD, Webb KA, O'Donnell DE (2005). Physiological changes during symptom recovery from moderate exacerbations of COPD. Eur Respir J.

[17] Hurst JR, Donaldson GC, Quint JK, Goldring JJ, Patel AR, Wedzicha JA (2010). Domiciliary pulse-oximetry at exacerbation of chronic obstructive pulmonary disease: prospective pilot study. BMC Pulm Med.

[18] Vanegas E, Igual R, Plaza I (2020). Sensing systems for respiration monitoring: a technical systematic review. Sensors (Basel).

[19] Chau JP, Lee DT, Yu DS, Chow AY, Yu W, Chair S, Lai ASF, Chick Y (2012). A feasibility study to investigate the acceptability and potential effectiveness of a telecare service for older people with chronic obstructive pulmonary disease. Int J Med Inform.

[20] Boiko A, Martínez Madrid Natividad, Seepold R (2023). Contactless technologies, sensors, and systems for cardiac and respiratory measurement during sleep: a systematic review. Sensors (Basel).

[21] Bujan B, Fischer T, Dietz-Terjung S, Bauerfeind A, Jedrysiak P, Große Sundrup Martina, Hamann J, Schöbel Christoph (2023). Clinical validation of a contactless respiration rate monitor. Sci Rep.

[22] Lauteslager T, Maslik M, Siddiqui F, Marfani S, Leschziner GD, Williams AJ (2021). Validation of a new contactless and continuous respiratory rate monitoring device based on ultra-wideband radar technology. Sensors (Basel).

[23] Borel J, Pelletier J, Taleux N, Briault A, Arnol N, Pison C, Tamisier R, Timsit J, Pepin J (2015). Parameters recorded by software of non-invasive ventilators predict COPD exacerbation: a proof-of-concept study. Thorax.

[24] Martín-Lesende Iñaki, Orruño Estibalitz, Bilbao A, Vergara I, Cairo MC, Bayón Juan Carlos, Reviriego E, Romo MI, Larrañaga Jesús, Asua J, Abad R, Recalde E (2013). Impact of telemonitoring home care patients with heart failure or chronic lung disease from primary care on healthcare resource use (the TELBIL study randomised controlled trial). BMC Health Serv Res.

[25] Yañez Aina M, Guerrero D, Pérez de Alejo Rigoberto, Garcia-Rio F, Alvarez-Sala JL, Calle-Rubio M, de Molina RM, Valle Falcones M, Ussetti P, Sauleda J, García Enrique Zamora, Rodríguez-González-Moro Jose Miguel, Franco Gay M, Torrent M, Agustí Alvar (2012). Monitoring breathing rate at home allows early identification of COPD exacerbations. Chest.

[26] Rubio N, Parker RA, Drost EM, Pinnock H, Weir CJ, Hanley J, Mantoani LC, MacNee W, McKinstry B, Rabinovich RA (2017). Home monitoring of breathing rate in people with chronic obstructive pulmonary disease: observational study of feasibility, acceptability, and change after exacerbation. COPD.

[27] Kranjec J, Beguš S, Geršak G, Drnovšek J (2014). Non-contact heart rate and heart rate variability measurements: a review. Biomedical Signal Processing and Control.

[28] van Gastel M, Stuijk S, Overeem S, van Dijk JP, van Gilst MM, de Haan G (2021). Camera-based vital signs monitoring during sleep - a proof of concept study. IEEE J Biomed Health Inform.

[29] Ichapurapu R, Jain S, John G, Monday T, Lie DYC, Banister R (2009). A 2.4GHz non-contact biosensor system for continuous vital-signs monitoring.

[30] Purnell L, Sierra M, Lisker S, Lim MS, Bailey E, Sarkar U, Lyles CR, Nguyen KH (2023). Acceptability and usability of a wearable device for sleep health among English- and Spanish-speaking patients in a safety net clinic: qualitative analysis. JMIR Form Res.

[31] World Medical Association (2013). World Medical Association Declaration of Helsinki: ethical principles for medical research involving human subjects. JAMA.

[32] (2020). Danish data protection legislation. Datatilsynet.

[33] Udsen FW, Lilholt PH, Hejlesen O, Ehlers LH (2014). Effectiveness and cost-effectiveness of telehealthcare for chronic obstructive pulmonary disease: study protocol for a cluster randomized controlled trial. Trials.

[34] Secher PH, Hangaard S, Kronborg T, Hæsum Lisa Korsbakke Emtekær, Udsen FW, Hejlesen O, Bender C (2022). Clinical implementation of an algorithm for predicting exacerbations in patients with COPD in telemonitoring: a study protocol for a single-blinded randomized controlled trial. Trials.

[35] Lungemedicin (2009). Hvordan vil du beskrive din KOL? Tag denne test [article in Danish]. Danish CATest.

[36] (2019). MRC-åndenødsskala. SYNLAB Medical Digital Services.

[37] Pelikan J, Ganahl K, Van den Broucke S, Sørensen K (2019). Measuring health literacy in Europe: Introducing the European Health Literacy Survey Questionnaire (HLS-EU-Q). International Handbook of Health Literacy.

[38] Ryser V, Meier C, Vilpert S, Maurer J (2023). Health literacy across personality traits among older adults: cross-sectional evidence from Switzerland. Eur J Ageing.

[39] Emtekaer Haesum Lisa Korsbakke, Ehlers L, Hejlesen OK (2015). Validation of the Test of Functional Health Literacy in Adults in a Danish population. Scand J Caring Sci.

[40] Rodriguez-Roisin R (2000). Toward a consensus definition for COPD exacerbations. Chest.

[41] Guest G, Bunce A, Johnson L (2006). How many interviews are enough?. Field Methods.

[42] Brooke J, Jordan PW, Thomas B, McClelland IL, Weerdmeester B (1996). SUS: A 'Quick and Dirty' Usability Scale. Usability Evaluation In Industry.

[43] Liamputtong P (2019). Handbook of Research Methods in Health Social Sciences.

[44] Braun V, Clarke V (2020). One size fits all? What counts as quality practice in (reflexive) thematic analysis?. Qual Res Psychol.

[45] Lewis JR, Sauro J (2017). Can I leave this one out? The effect of dropping an item from the SUS. J Usability Stud.

[46] Doyle C, Lennox L, Bell D (2013). A systematic review of evidence on the links between patient experience and clinical safety and effectiveness. BMJ Open.

[47] Garavand A, Aslani N, Nadri H, Abedini S, Dehghan S (2022). Acceptance of telemedicine technology among physicians: a systematic review. Inform Med Unlocked.

[48] Seemungal T A, Donaldson G C, Bhowmik A, Jeffries D J, Wedzicha J A (2000). Time course and recovery of exacerbations in patients with chronic obstructive pulmonary disease. Am J Respir Crit Care Med.

